# A Fusion Cytokine Coupling GMCSF to IL9 Induces Heterologous Receptor Clustering and STAT1 Hyperactivation through JAK2 Promiscuity

**DOI:** 10.1371/journal.pone.0069405

**Published:** 2013-07-01

**Authors:** Pingxin Li, Shala Yuan, Jacques Galipeau

**Affiliations:** 1 Department of Hematology and Medical Oncology, Winship Cancer Institute, Emory University, Atlanta, Georgia, United States of America; 2 Department of Pediatrics, Emory University, Atlanta, Georgia, United States of America; University of Medicine and Dentistry of New Jersey, United States of America

## Abstract

Cytokine receptors are randomly distributed on the cell surface membrane and are activated upon binding of their extracellular ligands to mediate downstream cellular activities. We hypothesized that pharmaceutical clustering of ligand-bound, activated receptors may lead to heretofore unrealized gain-of-function with therapeutically desirable properties. We here describe an engineered bifunctional cytokine borne of the fusion of Granulocyte Macrophage Colony Stimulating Factor (GMCSF) and Interleukin-9 (IL9) (hereafter GIFT9 fusokine) and demonstrate that it chaperones co-clustering of the functionally unrelated GMCSF receptor (GMCSFR) and IL9 receptor (IL9R) on cell surface of target cells. We demonstrate that GIFT9 treatment of MC/9 cells leads to transhyperphosphorylation of IL9R-associated STAT1 by GMCSFR-associated JAK2. We also show that IL9R-associated JAK1 and JAK3 augment phosphorylation of GMCSFR-linked STAT5. The functional relevance of these synergistic JAK/STAT transphosphorylation events translates to an increased mitogenic response by GMCSFR/IL9R-expressing primary marrow mast cells. The notion of inducing heterologous receptor clustering by engineered fusokines such as GIFT9 opens the door to a novel type of biopharmaceutical platform where designer fusokines modulate cell physiology through clustering of targeted receptor complexes.

## Introduction

It has been observed that cells exposed contemporaneously to distinct cytokines will cluster ligand-bound receptors to lipid rafts where they can influence each other’s downstream signaling [1,2]. This natural cooperativity observed in cytokine receptor biology leads us to speculate that pharmacologically impelled clustering of distinct ligand-activated receptors may lead to signal transduction synergies not otherwise observed *in vivo*. We have previously demonstrated that physical pairing of GMCSF with common γ chain (γc) interleukins as a single chain polypeptide leads to substantial gain-of-function properties when compared to the bioactivity of GMCSF and interleukins individually [3–5]. These GIFTs (GMCSF and Interleukin Fusion Transgene) typically lead to a STAT hyperphosphorylation response in stimulated lymphomyeloid cells [6], but the mechanism of the gain-of-function remains unclear.

The GMCSF receptor (GMCSFR) contains two subunits: GMCSFRα chain and the common β chain (βc) that is shared with IL3 and IL5 [7]. IL 9 receptor (IL9R) is constituted by IL9Rα chain and the common γ chain that is shared with other γc family interleukins including IL2, IL4, IL7, IL15, and IL21 [8,9]. Both GMCSFR and γc interleukin receptors signal through JAK/STAT pathways [10–12]. A total of 4 JAKs and 7 STATs have been identified to date [13]. The JAK family includes JAK1, JAK2, JAK3, and TYK2. They bind to the intracellular domains of receptors in an unphosphorylated form, and are rapidly self-phosphorylated and activated upon the extracellular binding of ligands [14]. The active forms of JAKs further phosphorylate specific tyrosine/serine residues on the receptors, leading the SH2-dependent recruitment of STATs, which are distributed in cellular cytoplasm as inactive homodimers [15]. After receptor binding, STATs are activated through phosphorylation by JAKs. Activated STATs dissociate from the receptors and translocate to cell nucleus to start transcription [16]. Each JAK associates with defined receptors and only activate specific STATs as their substrates. For instance, GMCSFR βc associates with JAK2, and utilize STAT5 as its substrate [17]. Unlike other JAKs that have broad expression patterns, the expression of JAK3 is restricted to leukocytes, and exclusively associates with interleukin γc receptors [18]. The association of IL9 with its receptor activates the phosphorylation of JAK1 and JAK3, which leads to the activation of STAT1, STAT3, and STAT5 [19].

Here, using a novel member of the GIFT family, the fusion of GMCSF and IL9 (GIFT9), we demonstrate that GIFT9 is able to induce the functional clustering of GMCSF and IL9 receptors and alter their respective downstream STAT phsophorylation signals through reciprocal JAK/STAT transactivation.

## Materials and Methods

### Ethics Statement

All animal experiments were approved by the Emory Institutional Animal Care and Use Committee and performed by accepted veterinary standards. C57BL/6 mice were sacrificed by CO_2_ and bone marrow cells were harvest from femurs and tibiae.

### Cell culture

JawsII cells were purchased from ATCC (Manassas, VA) and cultured in Alpha minimum essential medium (α-MEM) (Thermo Scientific, Waitham, MA) supplemented with 10% fetal bovine serum (Wisent Bioproducts, St. Bruno, Canada), 1% penicillin-streptomycin (Thermo Scientific), 1 mM sodium pyruvate (Thermo Scientific) and 5 ng/ml murine GMCSF (R & D systems, Minneapolis, MN) in a 5% CO_2_ incubator. MC/9 cells were purchased from ATCC and cultured in Dulbecco modified Eagle medium (DMEM) (Thermo Scientific) supplemented with 10% fetal bovine serum, 1% penicillin-streptomycin, 2 mM L-glutamine (Thermo Scientific), 0.05 mM 2-mercaptoethanol (Sigma, St. Louis, MO) and 10% Rat T-STIM (BD Biosciences, Franklin Lakes, NJ) in a 5% CO_2_ incubator. 293T cells were purchased from ATCC and cultured in DMEM supplemented with 10% fetal bovine serum, 1% Penicillin-streptomycin in a 5% CO_2_ incubator. 293-GP2 cells were purchased from Clontech and cultured in DMEM supplemented with 10% fetal bovine serum, 1% Penicillin-streptomycin in a 5% CO_2_ incubator. Recombinant mouse GMCSF and IL9 were purchased from R&D systems.

### Cloning and expression of GIFT9

Mouse GIFT9 cDNA was synthesized at GenScript USA Inc. (Piscataway, NJ) by aligning mouse GMCSF and IL9 cDNA. The GIFT9 cDNA was then cloned in a bicistronic retrovector AP-2 allowing the expression of GIFT9 and green fluorescent protein [4]. Infectious retroparticles were generated through transfection of 293-GP2 packaging cells (Clontech, Mountain View, CA) using PolyFect (Qiagen, Valencia, CA). Retroparticles were then used to transduce 293T cells. 293T cells were expanded, and the supernatant containing GIFT9 protein was collected and concentrated using Amicon centrifugation columns (Millipore, Billerica, MA). GIFT9 expression levels were quantified using mouse GMCSF ELISA kit (eBioscience, San Diego, CA).

### Cell treatment and Western blot

For cytokine stimulation assay in cell lines, cells were seeded at 9x10^6^/ml in 96-well plate, each well has 100 µl volume. Cells were starved in fetal bovine serum free medium for 5 hours with/without JAK inhibitors, and then were stimulated with 0.1 nM of GM-CSF, IL-9, both cytokines, and GIFT9 for 15 minutes at 37 °C. Cells were collected, wash once with ice-cold PBS, and lysed with lysis buffer supplemented with protease inhibitors and phosphatase inhibitor. Cell lysates were separated by SDS-PAGE, and Western blot analysis was performed with α-phosphorylated STAT1, STAT3, STAT5 antibodies (Cell signaling, Danvers, MA), total STAT1, STAT3, and STAT5 antibodies (cell Signaling) were used as a loading control. A representative result was shown from three independent experiments performed for each panel, the amount of STAT1 phosphorylation signal detected was quantified using the NIH ImageJ program and normalized against GMCSF+IL9 group. Student t-test was used to calculate the P-value. For MβCD treatment, 10 mg/ml MβCD (Sigma) was added to cells 1 hour before cytokine stimulation to disrupt lipid raft formation. JAK1, JAK2, and JAK3 inhibitors were purchased from Selleck Chemicals (Houston, TX) dissolved in DMSO, further diluted to desired concentration in PBS, and added to medium 5 hours before cytokine stimulation.

### Co-immunoprecipitation

For co-immunoprecipitation of GMCSF-Rβ by common γc receptor, MC/9 cells were stimulated with 1 nM of GM-CSF, IL-9, both cytokines, and GIFT9 for 15 minutes at 37 °C. After cytokine stimulation, MC/9 cells were washed twice with PBS and crosslinked with 0.5 mM DSP (Thermo Scientific) for 30 minutes at room temperature. The crosslinking was quenched by 25 mM Tris-HCl, pH 7.4 for 10 minutes. Cells were then washed again with ice-cold PBS and lysed with IP buffer (25 mM Tris-HCl, pH 7.4, 150 mM NaCl, 2 mM EDTA, 0.1% SDS, and 1% NP-40) by incubating on ice for 1 hour. Cell lysates were homogenized by passing through a 27-gauge needle at least 10 times. After removing cell debris by high-speed centrifugation, 1 mg protein from each sample was incubated with 2 mg of α-common γc antibody (Santa Cruz Biotechnology, Santa Cruz, CA) at 4 °C overnight. Next day sample-antibody complexes were incubated with 20 µl protein A agarose (Thermo Scientific) at 4 °C for 1.5 hour. Agarose was washed three times with IP buffer, reverse crosslink by incubating with 100 mM DTT in IP buffer at 37 °C for 1.5 hour. Samples were then boiled and separated by SDS-PAGE.

### Immunofluorescence staining and confocal microscopy

MC/9 cells were stimulated with 1 nM of GM-CSF, IL-9, both cytokines, and GIFT9 for 15 minutes at 37 °C. After cytokine stimulation, cells were spin to coverslip, fixed with 3.7% paraformaldehyde, and stained with α-GMCSF-Rβ (R&D systems) and α-common γc (Santa Cruz) antibodies at 4 °C overnight. Next day cells were probed with Donkey anti-rabbit Alexa 488 antibody and Donkey anti-goat Alexa 555 antibody (Invitrogen, Grand Island, NY) at room temperature for 2 hours. Coverslips were then mounted on slides with ProLong Gold Antifade Reagent with DAPI (Invitrogen). The images were captured using a Zeiss LSM 510 Confocal microscope at the integrated Cellular Imaging core of the Winship Cancer Institute at Emory University. For percentages of color pixel colocalization, 10 random selected cells from each group were analyzed by the ZEN software and graphed. P-value was calculated by student t-test.

### BMMC generation and MTT assay

BMMCs were generated essentially as previously described [20]. BMMCs were derived from 8-week-old C57BL/6J mice (The Jackson Laboratory, Bar Harbor, Maine). BMMCs were generated by culturing unfractionated C57BL/6 bone marrow cells with 5 ng/ml IL-3 and 50 ng/ml Stem Cell Factor (R&D systems) for 3 weeks. Mast cell phenotype was confirmed by flow cytometry analysis with α-c-kit (BD Biosciences) and α-FcεRIα (Biolegend, San Diego, CA) antibodies. BMMC cultures were greater than 97% mast cells at the time of use. MTT assay was performed essentially as previously described [21]. BMMCs were cultured with different cytokines for 5 days before analysis.

## Results

**Figure 1:**
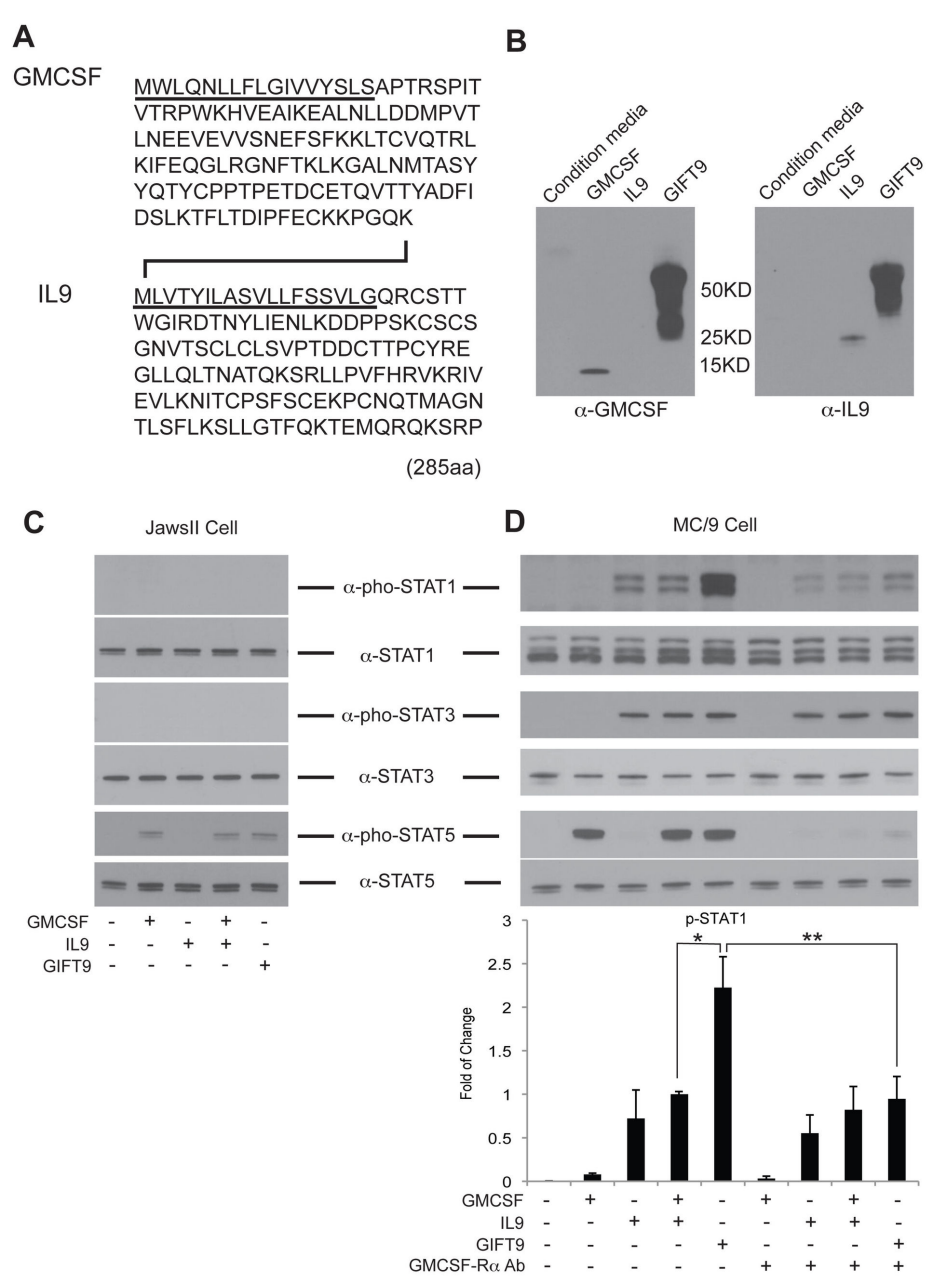
Mouse GIFT9 protein expression and biochemical analyses. (**A**) GIFT9 amino acid sequence. The signal peptides of each part are underscored, IL9 signal peptide serves as the linker of the two parts. (**B**) Western blot of GIFT9 protein in the condition media from 293T cells retrovirally transduced to express GIFT9. Recombinant mouse GMCSF and IL9 were used as controls. (**C**) Western Blot of phospho-STAT1, STAT3, and STAT5 in JawsII cells after GIFT9 stimulation. Total STAT protein was used as a loading control. (**D**) Western Blot of phospho-STAT1, STAT3, and STAT5 in MC/9 cells after GIFT9 stimulation without or with GMCSF-Rα antibody blocking. Total STAT protein was used as a loading control. Normalized STAT1 phosphorylation level is shown, *: P<0.05, **: P<0.01.

The GIFT9 fusion protein was created by aligning the cDNA encoding mouse GMCSF in frame with the 5’ end of the cDNA encoding mouse IL9. The stop codon of the mouse GMCSF cDNA was deleted, generating a cDNA encoding for a single 285 amino acid chain (Figure 1A). Western blots were performed using the conditioned media of 293T cells retrovirally transduced to express GIFT9. Our results show that both anti-GMCSF and anti-IL9 antibodies recognized the same secreted protein at a molecular weight of ~50 kD (Figure 1B). The molecular weight spread of GIFT9 protein observed on SDS gel electrophoresis are likely due to the glycosylation of the fusion protein, as it has been previously reported that both GMCSF and IL9 can be glycosylated at multiple sites [22,23].

To study the cellular biochemistry of GIFT9 on GMCSFR signaling, GMCSF responsive JawsII cells were stimulated by GIFT9 for 15 minutes and we observed that STAT5 phosphorylation levels were similar to that arising from either GMCSF alone or GMCSF and IL9 combination (Figure 1C). STAT1 and STAT3 phosphorylation levels remained undetectable as GMCSF is known to signal solely through JAK2-STAT5 pathway [7]. These data demonstrate that the GMCSF domain of GIFT9 is fully bioactive and deploys the canonical signaling properties of native GMCSF when bound to GMCSFR and that JawsII cells bereft of IL9R do not initiate γc interleukin driven STAT1/3 activation.

Upon treating GMCSFR^+^ IL9R^+^ MC/9 mast cells with GIFT9 for 15 minutes, a robust hyperphosphorylation of STAT1 was observed compared to controls, while phosphorylation of STAT3 and STAT5 remained similar (Figure 1D). Since MC/9 cells express both GMCSFR and IL9R, we blocked GMCSF-mediated signaling pathway by anti-GMCSFRα antibody to interrogate whether the hyperphosphorylation of STAT1 is solely due to the IL9/IL9R interaction. After blocking GMCSFRα, the hyperphosphorylation of STAT1 after GIFT9 stimulation was significantly decreased to a comparable level as IL9 stimulation alone (Figure 1D). These data suggest that the gain-of-function GIFT9-mediated hyperphosphorylation of STAT1 depends upon GMCSFR cooperativity with IL9R signaling pathways. Furthermore, the blocking of GMCSFR signaling pathway robustly suppressed STAT5 phosphorylation, reinforcing the role of GMCSFR in STAT5 activation in these cells.

**Figure 2:**
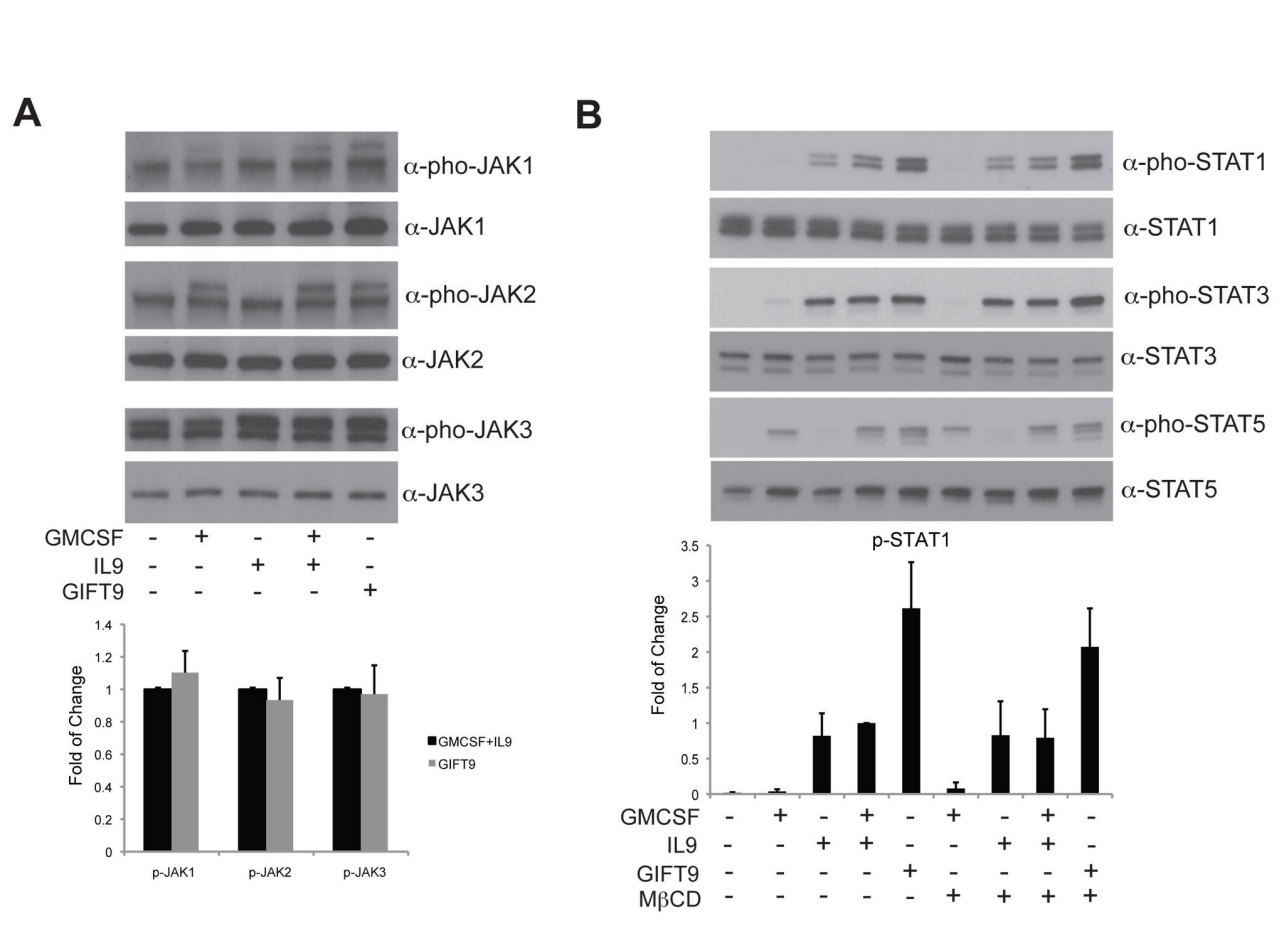
GIFT9 stimulation did not increase JAK kinase activity and receptor polarization to lipid rafts is not the major mechanism of GIFT9-induced hyperphosphorylation of STAT1. (**A**) JAK1, JAK2, and JAK3 phosphorylation levels remain same after GIFT9 stimulation. Total JAK protein was used as a loading control. Normalized JAK phosphorylation levels in GMCSF+IL9 and GIFT9 treatments are shown. (**B**) Inhibition of lipid rafts did not affect the hyperphosphorylation of STAT1 induced by GIFT9. Total STAT protein was used as a loading control. Normalized STAT1 phosphorylation level is shown.

Since STAT1 phosphorylation is dependent upon JAK1 kinase activity [18], our first hypothesis was that the hyperphosphorylation of STAT1 observed with GIFT9 stimulation of MC/9 cells was due to the hyperactivation of IL9R-associated JAK1. Western blot performed to examine the level of JAK1 phosphorylation after GIFT9 stimulation suggests that GIFT9 induced comparable levels of phosphorylated JAK1 as the combination treatment of GMCSF and IL9 (Figure 2A). Similar results were also obtained for JAK2 and JAK3 phosphorylation (Figure 2A), demonstrating that the hyperactivation of STAT1 was not due to hyperactivation of GMCSFR and IL9R associated JAK kinases.

Lipid rafts play an important role in supporting transphosphorylation between distinct cytokine receptors through receptor clustering [1,24]. Therefore, we hypothesized that lipid rafts may be important sites for recruiting GMCSFR and IL9R to induce hyperphosphorylation of STAT1 after GIFT9 treatment. To test this idea, MC/9 cells were pre-incubated with the lipid raft disruptor MβCD, followed by GIFT9 stimulation. Western blot analysis revealed that STAT1 phosphorylation after IL9 alone or GMCSF and IL9 double treatment remained similar after the disruption of lipid rafts (Figure 2B), suggesting that an alternate mechanism other than lipid raft polarization is involved in inducing GIFT9-mediated STAT1 hyperactivation.

**Figure 3:**
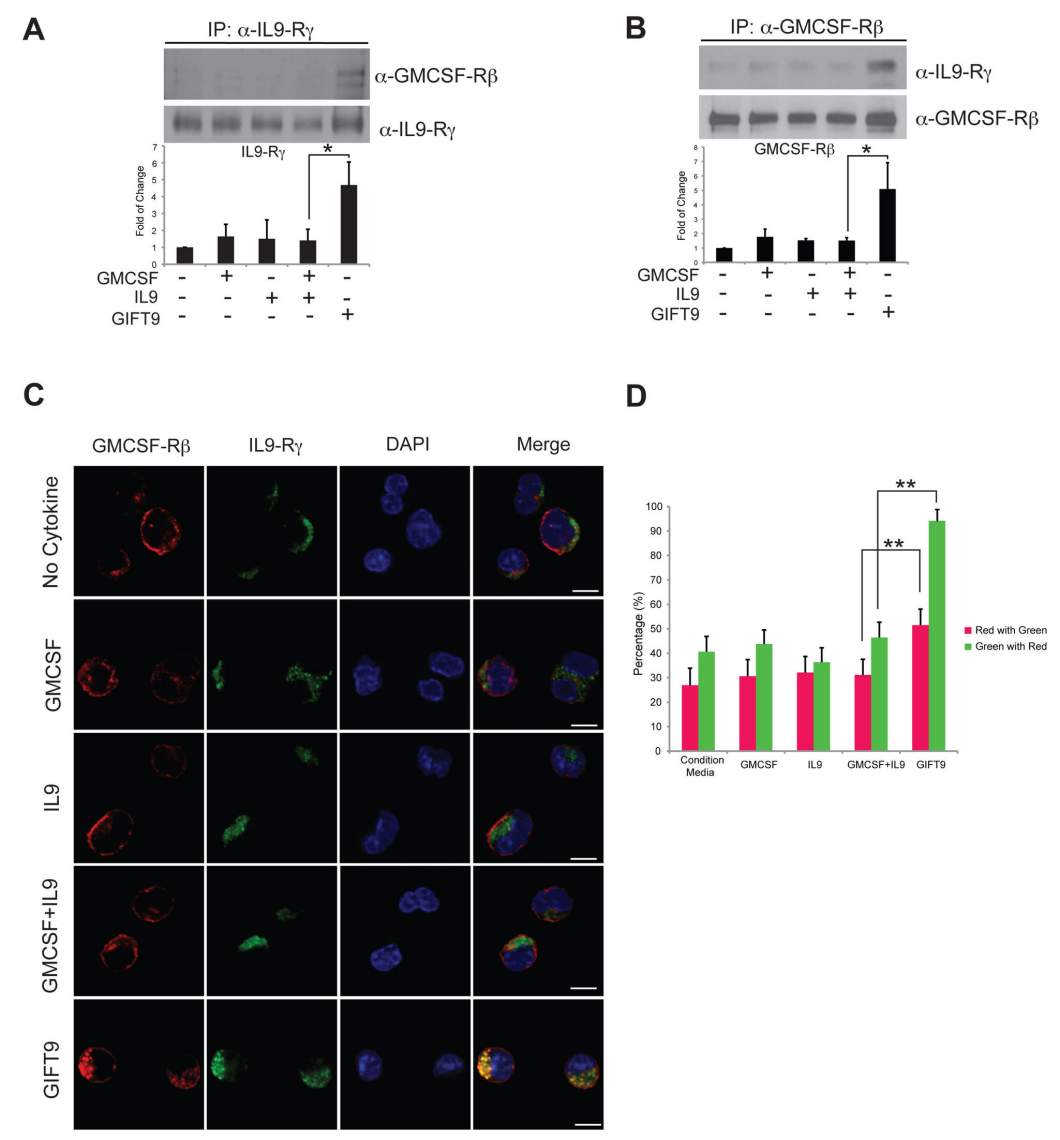
GMCSFR and IL9R colocalize after GIFT9 treatment. (**A**) GMCSFR βc was co-immunoprecipitated by α-common γc antibody after GIFT9 stimulation. *: P<0.05. (**B**) Common γc was co-immunoprecipitated by α-GMCSF-Rβ antibody after GIFT9 stimulation. *: P<0.05. (**C**) Representative confocal microscopy images of immunofluorescence staining showed the colocalization of GMCSF receptor and IL9 receptor after GIFT9 stimulation. Scale bar: 5 µm. (**D**) Percentages of color pixel colocalization after different cytokine treatment. **: P<0.01.

We further hypothesized that GIFT9 bioactivity may arise by its ability to chaperone the clustering of GMCSFR and IL9R independently of lipid raft polarization. To test this possibility, we first utilized a co-immunoprecipitation approach to test whether the IL9R γc and GMCSFR βc were physically associated when cells are treated with GIFT9. Following treatment of target MC/9 cells with GIFT9, we immunoprecipitated the interleukin γc and its associated proteins and subsequently probed for GMCSFR βc by Western blot. The co-immunoprecipitation assay showed that a significantly increased amount of GMCSFR βc was pulled down with IL9 γc after GIFT9 treatment (Figure 3A), while only background level of GMCSFR βc was pulled down by IL9R γc after single or double cytokine treatment. These data suggest that GIFT9 chaperones the clustering of GMCSFR and IL9R into a stable complex. The reciprocal co-immunoprecipitation experiment was also performed, and similar results were observed (Figure 3B).

To provide further evidence to support the hypothesis of GIFT9-mediated clustering of GMCSFR and IL9R, MC/9 cells were double stained with GMCSFR and IL9R antibodies after cytokine treatments and immunofluorescent images were visualized by confocal microscopy. After single or double GMCSF and IL9 treatment, GMCSFR and IL9R were not meaningfully co-localized, whilst receptor co-localization was evident after GIFT9 treatment (Figure 3C). Analyses of individual cells in each treatment group demonstrated that the percentage of color pixel co-localization are significantly higher, approaching unity, in the GIFT9-treated group comparing with controls (Figure 3D).

**Figure 4:**
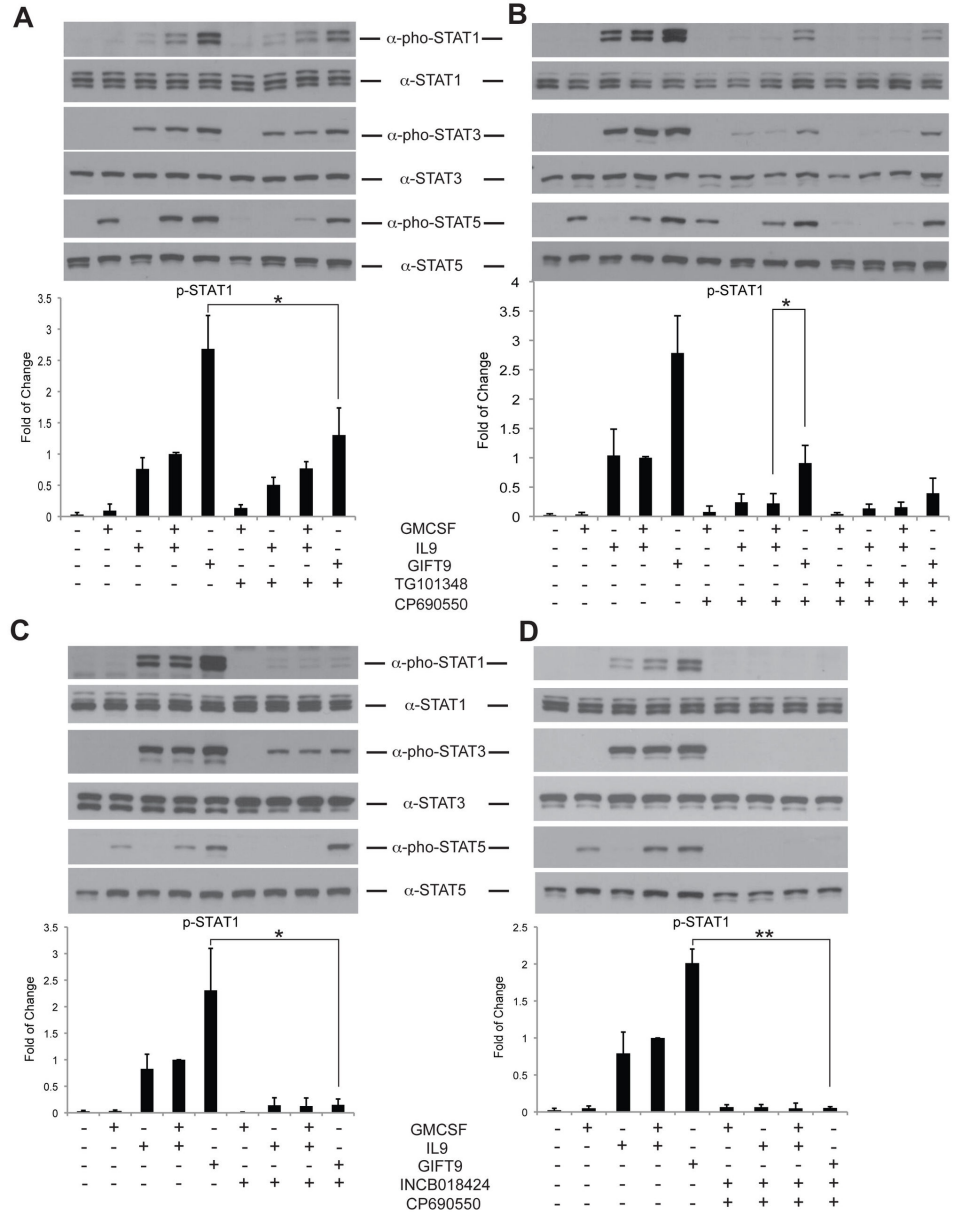
JAK2 transphosphorylates STAT1 after GIFT9 treatment. (**A**) JAK2 inhibitor TG101348 treatment abolished hyperphosphorylation of STAT1 after GIFT9 stimulation. Total STAT protein was used as a loading control. (**B**) JAK3 inhibitor CP690550 treatment had a minor effect of STAT1 hyperphosphorylation induced by JAK2. Total STAT protein was used as a loading control. (**C**) Double inhibition of JAK1 and JAK2 by INCB018424 inhibited STAT5 phosphorylation after GMCSF/IL9 but not GIFT9 stimulation. Total STAT protein was used as a loading control. (**D**) Inhibition of all JAKs by INCB018424 and CP690550 depleted STATs phosphorylation. Total STAT protein was used as a loading control. Normalized STAT1 phosphorylation level is shown, *: P<0.05, **: P<0.01.

The physical evidence of GIFT9-mediated co-localization of GMCSFR and IL9R provides a testable explanation for the observed hyperphosphorylation of IL9R-associated STAT1 in MC/9 cells. STAT1 hyperactivation may be due to transphophorylation by promiscuous JAK2 activity that is normally associated with GMCSFR. Indeed, STAT1 is known to be a JAK2 target in other circumstances [25,26]. To test this idea, MC/9 cells were pre-treated with the JAK2 inhibitor TG101348 for 5 hours and STAT activation levels were examined. After JAK2 inhibition, GIFT9 induced STAT1 phosphorylation decreased to a comparable level as GMCSF and IL9 double treatment, while STAT3 phosphorylation remained unchanged (Figure 4A). These data strongly support the theory that GMCSFR associated JAK2 transphosphorylates IL9R-associated STAT1. Interestingly, STAT5 phosphorylation levels were nearly abolished in single and double cytokine treated samples but not in GIFT9 treated cells (Figure 4A), suggesting that other JAKs may deploy STAT5 tropism when dominant JAK2 is blocked. To test whether other JAKs are involved in transphosphorylation of STAT5, we first inhibited JAK3 function by pre-incubating MC/9 cells with the JAK3 inhibitor CP690550. Following JAK3 inhibition, STAT1 phosphorylation decreased after GIFT9 and abolished STAT1 activation was observed in GMCSF and GMCSF+ IL9 double treatment groups (Figure 4B). STAT3 phosphorylation decreased as well, which is consistent with previous studies as JAK3-STAT3 signals through the IL9R γc. These data suggested that JAK3 phosphorylates STAT1 in a canonical response to IL9. However, GIFT9 provides a redundant STAT1 activation pathway afforded by JAK2. To validate the hypothesis that GIFT9 leads to STAT1 hyperphosphorylation through the activities of both JAK2 and JAK3, we inhibited JAK2 and JAK3 activities jointly by the use of both TG101348 and CP690550 simultaneously. Dual JAK2/JAK3 inhibition abolished STAT1/3/5 activation in MC/9 cells following treatment with GMCSF+IL9, yet meaningful activation of all STATs was still apparent following GIFT9 treatment (Figure 4B). These data suggest a possible role for JAK1 kinase activity deployed by GIFT9 activation. To interrogate JAK1 activity, we used the JAK1/2 inhibitor INCB018424 to treat the MC/9 cells before cytokine stimulation. Western blot showed that when both JAK1 and JAK2 were inhibited, STAT1 phosphorylation reduced to basal levels and STAT3 phosphorylation decreased modestly, likely reflecting residual JAK3 activity (Figure 4C). Since STAT1/3/5 phosphorylation persists after GIFT9 treatment despite JAK2 and JAK3 co-inhibition, we inhibited all three JAKs by using INCB018424 and CP690550 simultaneously to determine whether other non-canonical kinases contributed to STAT phosphorylation in response to GIFT9. Western blot analysis reveals that GIFT9-mediated STAT1, STAT3 and STAT5 phosphorylation were completely abolished when JAK1/2/3 were inhibited contemporaneously (Figure 4D), confirming that virtually all the GIFT9-mediated gain-of-function STAT phosphorylation events are mediated solely through GMCSFR and IL9R associated JAK1/2/3.

**Figure 5:**
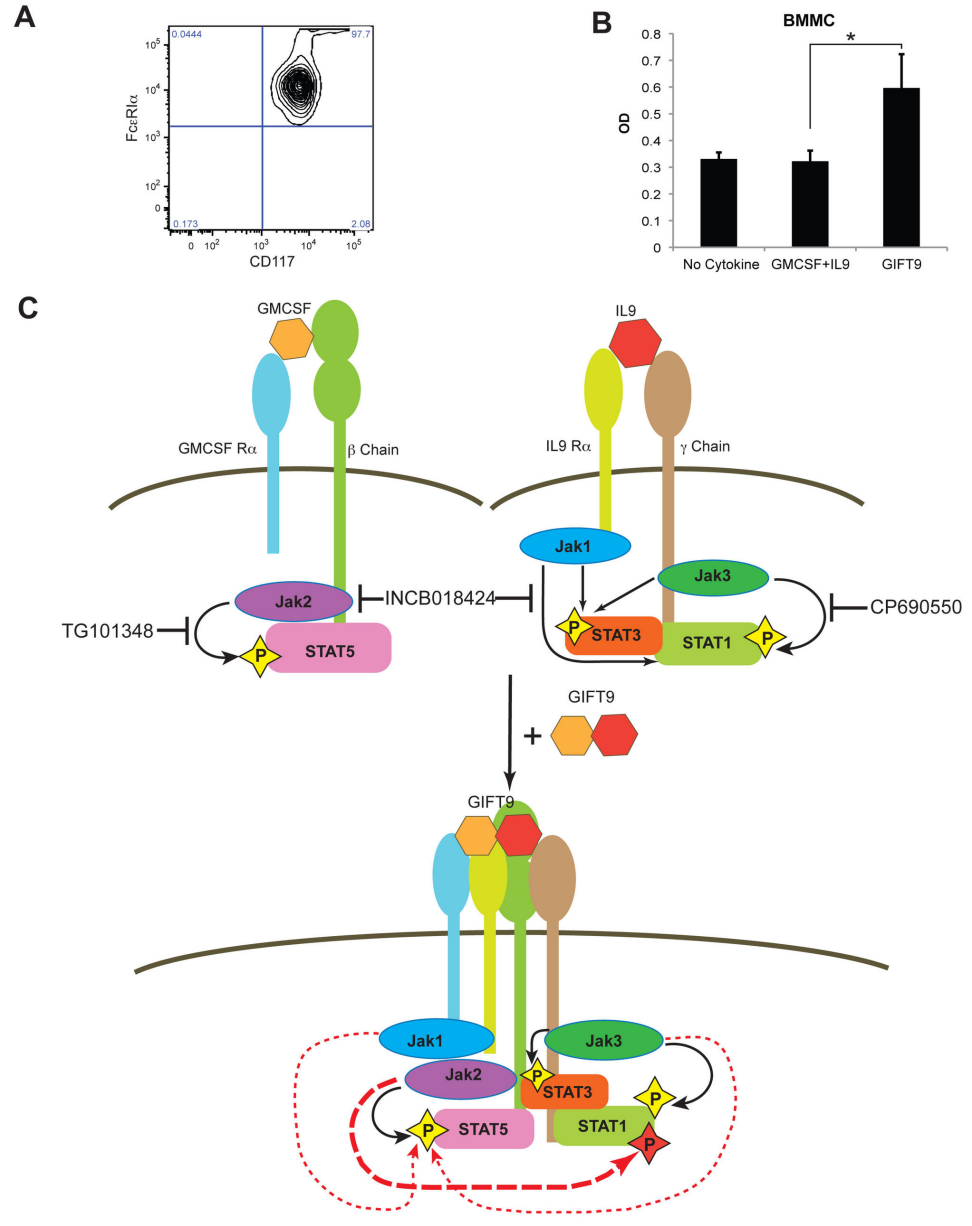
GIFT9 stimulates the growth of BMMCs. (**A**) Flow cytometry analysis of BMMCs. (**B**) MTT assay of BMMCs after 5 days of culture with GMCSF/IL9 or GIFT9. *: P<0.05. (**C**) Model of GMCSF and IL9 receptor clustering and downstream signaling after GIFT9 stimulation. See text for further details.

It is known that CD117^+^ FcεRIα^+^ mast cells are IL9-responsive [27]. To validate whether GIFT9-mediated receptor clustering leads to meaningful gain of function in non-transformed normal myeloid cells, we tested whether bone marrow derived mast cells deployed an alternate functionality following exposure to GIFT9 *in vitro*. We harvested unfractionated bone marrow cells from C57BL/6J mice and culture expanded primary mast cells with IL-3 and Stem Cell Factor [20]. Flow cytometry analysis showed that bone marrow mast cell cultures had over 97% mast cells at the time of use (Figure 5A). We subsequently treated mast cells with GIFT9 and compared proliferation rate to controls and found that mast cells treated with GIFT9 significantly increased their proliferative rate by 100% (Figure 5B).

## Discussion

In the current study we present a novel engineered biopharmaceutical platform – fusokines – that leads to heterogeneous cytokine receptor clustering, transactivation and acquisition of heretofore unheralded cell biochemical responses. Using GIFT9 as a proof of concept, we show that the gain-of-function induced by GIFT9 treatment is due to the physical clustering of GMCSFR and IL9R with GIFT9 behaving as an extracellular activator/chaperone. We demonstrated that GIFT9 leads to hyperphosphorylation of STAT1, with acquisition of redundant yet homeostatic STAT3 and STAT5 phosphorylation levels. We further found that the GIFT9-mediated hyperphosphorylation of STAT1 is neither due to the hyperactivation of JAKs, nor the polarization of GMCSFR and IL9R to membrane lipid rafts. Rather, we found that GMCSFR/IL9R clustering enables GMCSFR-associated JAK2 to transphosphorylate IL9R-associated STAT1. Different from STAT1 phosphorylation, STAT3 phosphorylation level remains similar after GIFT9 treatment although JAK2 has been previously reported to be able to phophorylate STAT3 [28]. This could be due to that STAT3 phosphorylation already reaches its maximum level driven by JAK1/3, as STAT3 phosphorylation remained unchanged when JAK2 was inhibited but decreased when JAK3 were inhibited after GIFT9 treatment (Figure 4A-B). Interestingly, STAT5 phosphorylation levels remained high after GIFT9 treatment when JAK2 kinase function is inhibited. Further experiments demonstrated that receptor clustering deployed IL9R-associated JAK1 and JAK3 kinase activities targeting GMCSFR-associated STAT5 (Figure 5C).

Under physiological circumstances, unrelated cytokine receptors are randomly distributed on cell membrane surface. When ligand bound, individual receptors can migrate to lipid rafts where they may interact with each other [1,24]. This may represent a mechanism by which a cell can integrate synergistically the signals mediated by its cytokine milieu. In an effort to translate this biological insight in to a medically useful therapeutic strategy, we here tested a synthetic bi-domain cytokine and its ability to act as a chaperone and mediate receptor clustering and synergistic gain-of-function properties. The development of fusion cytokine proteins is potentially of importance since it provides a novel strategy to bring together two (or even more) receptors that are usually not in the same signaling complex, so as to trigger novel downstream events that cannot be achieved by any other methods. This platform concept is entirely distinct in both form and function to the use of small molecule agonists, recombinant cytokine monotherapy or the use of targeted monoclonal antibodies [29,30]. Furthermore, the fusokine chaperone effect bridges activated receptors, a property which cannot be replicated by simple apposition of inactive cell surface receptors by monoclonal antibodies even if they were to be “bi-specific” [31]. As demonstrated here by the GIFT9 prototype, the design of fusion proteins can be extended in future to hypothesis-driven cytokine coupling based on their targeting cell’s receptor expression pattern in order to elicit target specific gain-of-function pharmaceutical effects.
